# Effectiveness of Vasopressin Against Cardiac Arrest: A Systematic Review of Systematic Reviews

**DOI:** 10.1007/s10557-024-07571-3

**Published:** 2024-03-12

**Authors:** Jonathan Ka-Ming Ho, Hon-Lon Tam, Leona Yuen-Ling Leung

**Affiliations:** 1 School of Nursing and Health Studies, Hong Kong Metropolitan University, Homantin, Kowloon, Hong Kong; 2 The Nethersole School of Nursing, The Chinese University of Hong Kong, Shatin, New Territories Hong Kong

**Keywords:** Cardiopulmonary resuscitation, Epinephrine, Heart arrest, Heart massage, Steroids, Vasopressins

## Abstract

**Purpose:**

This systematic review (SR) of SRs evaluates the effectiveness of vasopressin alone or in combination with other drugs in improving the outcomes of cardiac arrest (CA).

**Methods:**

Using a three-step approach, we searched five databases to identify all relevant SRs. Two reviewers independently selected suitable studies, assessed study quality, and extracted relevant data. If an outcome was reported by multiple SRs, a re-meta-analysis was conducted as needed; otherwise, a narrative analysis was performed.

**Results:**

Twelve SRs covering 16 original studies were included in this review. The meta-analysis results revealed a significant increase in survival to hospital admission for patients with in-hospital CA (IHCA) or out-of-hospital CA (OHCA) receiving vasopressin alone compared with that for those receiving epinephrine alone. Furthermore, the return of spontaneous circulation (ROSC) was significantly increased in patients with OHCA receiving vasopressin with epinephrine compared with that in those receiving epinephrine alone. Compared with patients with IHCA receiving epinephrine with placebo, those receiving vasopressin, steroids, and epinephrine (VSE) exhibited significant increases in ROSC, survival to hospital discharge, favorable neurological outcomes, mean arterial pressure, renal failure–free days, coagulation failure–free days, and insulin requirement.

**Conclusion:**

VSE is the most effective drug combination for improving the short- and long-term outcomes of IHCA. It is recommended to use VSE in patients with IHCA. Future studies should investigate the effectiveness of VSE against OHCA and CA of various etiologies, the types and standard dosages of steroids for cardiac resuscitation, and the effectiveness of vasopressin–steroid in improving CA outcomes.

**Supplementary Information:**

The online version contains supplementary material available at 10.1007/s10557-024-07571-3.

## Introduction

Cardiac arrest (CA) refers to an abrupt interruption of blood flow to the brain and other organs because of the ineffective pumping of the heart. This medical emergency is a common cause of mortality worldwide [1]. A systematic review (SR) of 67 studies indicated that the global incidence of out-of-hospital CA (OHCA) in adults was 55 cases per 100,000 person-years [2]. A meta-analysis of 141 studies revealed that the pooled rates of survival to hospital admission (STHA) and survival to hospital discharge (STHD) in patients with OHCA were 22% and 9%, respectively [3]. However, the global incidence of in-hospital CA (IHCA) and the associated rates of patient survival remain somewhat unclear. In a review of relevant studies conducted in the United States, the annual number of adult IHCA cases was reported to be more than 290,000, and the rate of STHD was reported to be 25% [4]. In addition to cardiopulmonary resuscitation (CPR) and early defibrillation, pharmacological therapy is crucial for ensuring the return of spontaneous circulation (ROSC) and thereby saving the life of patients with CA [1].

Epinephrine has been used as the standard vasopressor for several decades [5, 6]. This drug enhances vascular tone, heart rate, and cardiac contractility to increase mean arterial pressure (MAP) and coronary perfusion pressure, thus improving coronary blood flow and facilitating ROSC [7]. However, the adrenergic effects of epinephrine increase myocardial oxygen consumption and may lead to myocardial dysfunction, which is associated with poor hemodynamic and neurological outcomes [8]. This prompted researchers to explore other drugs for improving CA outcomes [9].

In the late 1990s, vasopressin was proposed as an alternative or adjunct to epinephrine for cardiac resuscitation [10]. Vasopressin increases MAP and coronary perfusion pressure by enhancing vascular tone and thus accelerating ROSC by increasing coronary blood flow [11]. Unlike epinephrine, vasopressin does not increase myocardial oxygen consumption because it does not exert chronotropic or inotropic effects [12]. Nevertheless, both vasopressors are effective in improving the short-term outcomes of CA, such as ROSC and STHA, but not the long-term outcomes of CA, such as STHD and favorable neurological outcomes (FNO) [13].

Steroids have been recommended for use in combination with epinephrine and vasopressin for CA treatment. Studies have reported that ischemic injury of the hypothalamic–pituitary–adrenal axis leads to adrenal insufficiency during and after cardiac resuscitation and a reduction in the serum cortisol level, which is associated with reductions in ROSC and STHD [14, 15]. Theoretically, the administration of steroids during and after cardiac resuscitation can restore the serum cortisol level, and therefore, steroids can simultaneously improve the short- and long-term outcomes of CA [16, 17].

Whether vasopressin should be used alone or in combination with other drugs to improve CA outcomes remains a topic of debate. Although multiple studies and reviews have focused on this topic, their findings have been inconsistent. Since no SR of SRs has been conducted on this topic, we conducted the present review to synthesize evidence related to the effectiveness of vasopressin alone or in combination with other drugs in improving CA outcomes.

## Methods

The protocol of this review has been registered with the International Prospective Register of Systematic Reviews (registration number: CRD42022334077). The essential components of this review were identified on the basis of the Preferred Reporting Items for Systematic Reviews and Meta-Analyses (PRISMA) 2020 guidelines [18]. The PRISMA 2020 checklist is presented in Supplementary Information 1.

### Eligibility Criteria

#### Population

This review included SRs focusing on adult patients (age ≥ 18 years) with IHCA or OHCA. SRs focusing on animals and patients with traumatic CA were excluded from this review.

#### Intervention

The intervention was the administration of vasopressin alone or in combination with other drugs.

#### Comparator

The main comparators were placebo and nonvasopressin drugs, such as epinephrine. Vasopressin alone was compared with its combination with other drugs, and vice versa.

#### Outcomes

The primary outcome was ROSC (i.e., restoration of sustained cardiac activity with significant respiratory effort). The secondary outcomes were STHA (i.e., maintenance of spontaneous circulation upon admission to the hospital), STHD (i.e., maintenance of spontaneous circulation at discharge from the hospital), FNO (i.e., Glasgow–Pittsburgh Cerebral Performance Category 1 or 2), and others.

#### Study Design

SRs with or without meta-analysis were eligible for this review.

### Search Strategy

The Cochrane Library, MEDLINE, ProQuest Health and Medical Collection, Scopus, and Web of Science databases were searched to identify potentially eligible SRs published in English. No restriction was imposed regarding publication year. A three-step approach was adopted for the literature search. First, the electronic database MEDLINE was searched to identify keywords included in the title or abstract and index terms. Second, all electronic databases were extensively searched using all identified keywords and index terms. Third, the reference lists of all identified studies were manually searched to identify relevant SRs. The search strategies for all databases are illustrated in Supplementary Information 2.

### Study Selection

The search results were imported to Rayyan, which is a free Web and mobile app for screening the studies for SRs [19]. After removing duplicate results, two reviewers (JKM and HL) independently screened the titles and abstracts or even full text of relevant studies to evaluate their eligibility for this review. Any disagreements between the two reviewers were resolved with a third reviewer (LYL) through discussion.

### Quality Assessment

Two reviewers (JKM and HL) independently assessed the quality, including the risk of bias (RoB), of the included SRs. The assessment was performed using A MeaSurement Tool to Access systematic Reviews 2 (AMSTAR 2) [20]. Any disagreements between the two reviewers were resolved with the third reviewer (LYL) through discussion. The details of AMSTAR 2 are presented in Supplementary Information 3.

### Data Extraction

Two independent reviewers (JKM and HL) extracted relevant data from the included SRs by using a self-developed data extraction form (Microsoft Excel). The data comprised publication details, study settings, study populations, inclusion and exclusion criteria, sample sizes, interventions and comparators, outcome measures, study results, and authors’ conclusions. Any discrepancies between the two reviewers were resolved with the third reviewer (LYL) through discussion.

### Data Synthesis

If an outcome was reported by multiple SRs, a re-meta-analysis was conducted to estimate the effects of vasopressin on the outcome as needed [21]. After the removal of duplicate studies from the included SRs, the freeware Review Manager (version 5.4) was used to pool the data of the original studies after ensuring a lack of clinical heterogeneity in terms of the study settings, study populations, interventions and comparators, and outcome measures. Odds ratios (ORs) with 95% confidence intervals (CIs) were calculated. Statistical heterogeneity was assessed using the *I*^2^ test. A fixed-effects model was adopted if the *I*^2^ value was ≤ 50%, and a random-effects model was adopted if this value was > 50% [22]. A narrative analysis was performed if an outcome was reported by only one SR or if a re-meta-analysis was unnecessary (e.g., all original studies were included in a previous meta-analysis).

### Protocol Deviation

This review adhered to the registered protocol without any changes.

## Results

### Study Retrieval and Selection

**Fig. 1:**
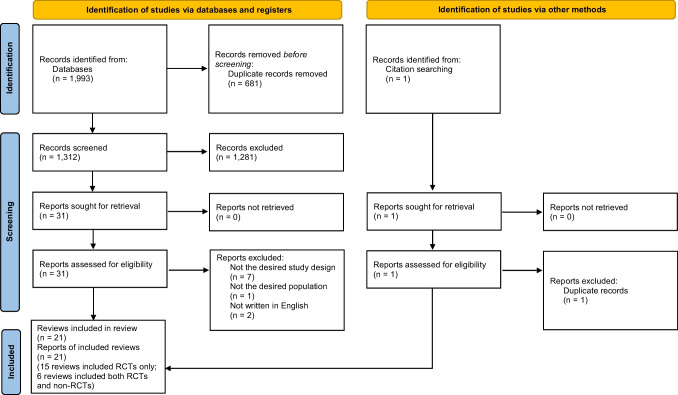
Flow diagram for the study retrieval and selection process

Figure 1 illustrates the flow diagram for the study retrieval and selection process. Supplementary Information 4 presents a list of studies excluded after a full-text review. The literature was searched between May 18 and 25, 2022, and the search was performed again between August 13 and 15, 2023 when finalizing this review. A total of 1,993 articles were identified, and 21 SRs were eligible for this review [9, 13, 16, 17, 23–39].

### Study Quality

Table 1 summarizes the AMSTAR 2 results of the 21 eligible SRs. The AMSTAR 2 assessment revealed that only 1 SR had high quality [13], 11 had low quality [9, 16, 17, 26, 28, 33–35, 37–39], and 9 had critically low quality [23–25, 27, 29–32, 36]. To ensure the quality of evidence, we excluded the SRs with critically low quality. Subsequently, 12 SRs [9, 13, 16, 17, 26, 28, 33–35, 37–39] were included in this review. Despite the exclusion of the SRs with critically low quality, the included SRs covered all 16 original studies [40–55]. Notably, the included SRs indicated that most of the original studies had a low RoB [9, 13, 16, 17, 26, 28, 33–35, 37–39].

**Table 1:** AMSTAR 2 results of the eligible systematic reviews

Citation	Item																		Quality
	**1**	**2**†	**3**	**4**†	**5**	**6**	**7**†	**8**	**9**†		**10**	**11**†		**12**	**13**†	**14**	**15**†	**16**	
									**RCT**	**NRSI**		**RCT**	**NRSI**						
Abdelazeem et al. (2022)	Y	Y	N	PY	Y	Y	N	PY	Y	NA	N	Y	NA	Y	Y	Y	Y	Y	L
An et al. (2022)	N	PY	N	PY	Y	Y	N	N	N	N	N	Y	N	N	Y	N	Y	Y	CL
Aung & Htay (2005)	Y	PY	N	PY	Y	Y	N	Y	PY	NA	N	Y	NA	N	N	Y	Y	Y	CL
Aves et al. (2020)	Y	PY	N	PY	Y	Y	N	Y	Y	NA	N	Y	NA	N	N	N	Y	N	CL
Belletti et al. (2018)	Y	PY	N	PY	Y	Y	Y	PY	Y	NA	N	Y	NA	N	Y	Y	N	N	L
Biondi-Zoccai et al. (2003)	Y	N	N	N	N	N	N	N	N	NA	N	N	NA	N	N	Y	Y	N	CL
Finn et al. (2019)	Y	Y	N	PY	Y	Y	Y	PY	Y	NA	Y	Y	NA	Y	Y	Y	Y	Y	H
Holmberg et al. (2019)	Y	Y	N	PY	Y	Y	N	PY	Y	Y	N	Y	N	N	N	N	Y	Y	CL
Holmberg et al. (2022)	Y	Y	N	PY	Y	Y	N	PY	Y	NA	N	Y	NA	Y	Y	Y	Y	Y	L
Jing et al. (2010)	Y	N	N	PY	N	N	N	PY	N	N	N	Y	N	N	N	Y	Y	Y	CL
Larabee et al. (2012)	Y	N	N	PY	N	N	N	N	N	N	N	NA	NA	NA	N	N	NA	Y	CL
Layek et al. (2014)	Y	PY	N	PY	Y	Y	N	N	Y	NA	N	Y	NA	Y	N	N	Y	Y	CL
Li et al. (2020)	Y	PY	N	PY	Y	Y	N	PY	Y	NA	N	NA	NA	NA	Y	Y	NA	Y	L
Lin et al. (2014)	Y	PY	N	PY	Y	Y	Y	N	Y	NA	N	Y	NA	N	N	Y	Y	Y	L
Mentzelopoulos et al. (2012)	Y	PY	N	PY	Y	Y	N	PY	Y	NA	N	Y	NA	Y	Y	Y	Y	Y	L
Morales-Cane et al. (2016)	Y	N	N	PY	N	N	N	N	N	N	N	Y	N	N	Y	N	Y	N	CL
Saghafi et al. (2022)	Y	PY	N	PY	Y	Y	N	N	Y	NA	N	Y	NA	Y	Y	Y	Y	Y	L
Satti et al. (2022)	Y	Y	N	PY	N	Y	N	Y	Y	NA	N	Y	NA	Y	Y	Y	NA	Y	L
Shah & Mitra (2021)	Y	Y	N	PY	Y	Y	N	PY	Y	Y	N	Y	Y	N	Y	Y	Y	Y	L
Sillberg et al. (2008)	Y	PY	N	PY	Y	Y	N	PY	PY	NA	N	NA	NA	NA	Y	N	NA	Y	L
Zhang et al. (2017)	Y	PY	N	PY	Y	Y	N	PY	Y	NA	N	Y	NA	Y	Y	Y	Y	Y	L

^†^ Critical domains

Abbreviations: CL, critically low; H, high; L, low; N, no; NA, not applicable; NRSI, non-randomized study of intervention; PY, partial yes; RCT, randomized controlled trial; Y, yes

Notes:

Item 1 – Did the research questions and inclusion criteria for the review include the components of PICO (population, intervention, comparator group, outcome)?

Item 2 – Did the report of the review contain an explicit statement that the review methods were established prior to the conduct of the review and did the report justify any significant deviations from the protocol?

Item 3 – Did the review authors explain their selection of the study designs for inclusion in the review?

Item 4 – Did the review authors use a comprehensive literature search strategy?

Item 5 – Did the review authors perform study selection in duplicate?

Item 6 – Did the review authors perform data extraction in duplicate?

Item 7 – Did the review authors provide a list of excluded studies and justify the exclusions?

Item 8 – Did the review authors describe the included studies in adequate detail?

Item 9 – Did the review authors use a satisfactory technique for assessing the risk of bias (RoB) in individual studies that were included in the review?

Item 10 – Did the review authors report on the sources of funding for the studies included in the review?

Item 11 – If meta-analysis was performed, did the review authors use appropriate methods for statistical combination of results?

Item 12 – If meta-analysis was performed, did the review authors assess the potential impact of RoB in individual studies on the results of the meta-analysis or other evidence synthesis?

Item 13 – Did the review authors account for RoB in individual studies when interpreting/ discussing the results of the review?

Item 14 – Did the review authors provide a satisfactory explanation for, and discussion of, any heterogeneity observed in the results of the review?

Item 15 – If they performed quantitative synthesis, did the review authors carry out an adequate investigation of publication bias (small study bias) and discuss its likely impact on the results of the review?

Item 16 – Did the review authors report any potential sources of conflict of interest, including any funding they received for conducting the review?

### Study Characteristics

Table 2 presents the characteristics of the included SRs, and Table 3 lists the original studies reviewed in each SR. It is necessary to mention that all drugs were administered intravenously. Vasopressin was administered at a dosage of 40 IU per CPR cycle (1 dose or 2 doses) for a comparison of vasopressin alone or vasopressin–epinephrine with epinephrine alone and at a dosage of 20 IU per CPR cycle (4–5 doses) for a comparison of VSE with epinephrine–placebo. Epinephrine was administered at a dosage of 1 mg per CPR cycle. Methylprednisolone was administered at a dosage of 40 mg during CPR with or without hydrocortisone administered at a dosage of 300 mg for 7 days [9, 13, 16, 17, 26, 28, 33–35, 37–39].

**Table 2:** Characteristics of the included systematic reviews

Citation	Aim	Search Strategy	Studies and Participants	PICOS	RoB	Authors’ Conclusion
Abdelazeem et al. (2022)	To evaluate the effects of combining VSE for ROSC and STHD in IHCA patients	Database: PubMed, Embase, Scopus, Web of Science, Cochrane Central, and Google Scholar Period: From inception to 17 October 2021 Language: English Search term: Defined Reference tracking or other sources: Yes	- 3 RCTs - 869 participants	Population: IHCA patients Intervention: VSE Comparator: Placebo and epinephrine Outcome: ROSC, STHD, and FNO Study design: RCTs	Complete and satisfactory quality assessment of the included studies	The usage of VSE was associated with increased ROSC compared with placebo and epinephrine, but there was no difference regarding STHD
Belletti et al. (2018)	To compare all the vasopressors tested in RCTs in CA adult patients in order to identify the treatment associated with the highest rate of ROSC, survival, and FNO	Database: PubMed, Embase, BioMed Central, and Cochrane Central Register Period: From inception to 1 April 2017 Language: Not mentioned Search term: Defined Reference tracking or other sources: Yes	- 28 RCTs - 14,848 participants	Population: IHCA and OHCA patients Intervention and comparator (Network meta-analysis): Placebo; low-dose epinephrine; high-dose epinephrine; norepinephrine; lidocaine; methoxamine; phenylephrine; isoproterenol and epinephrine; vasopressin; vasopressin and epinephrine; VSE Outcome: ROSC, FNO, and survival at the longest follow-up Study design: RCTs	Complete and satisfactory quality assessment of the included studies	Only a combination of VSE was associated with improved survival with FNO and ROSC probability compared with several other comparators, particularly in IHCA
Finn et al. (2019)	To determine whether epinephrine or vasopressin or both, administered during CA, afford any survival benefit	Database: Cochrane Central Register of Controlled Trials, Database of Abstracts of Reviews of Effects, MEDLINE, and Embase Period: From inception to 8 May 2018 Language: Not limited Search term: Defined Reference tracking or other sources: Yes	- 26 RCTs - 21,704 participants	Population: IHCA and OHCA patients Intervention: Standard-dose epinephrine Comparator: Placebo; high-dose epinephrine; vasopressin; vasopressin and epinephrine Outcome:: ROSC, STHA, STHD, and FNO Study design: RCTs	Complete and satisfactory quality assessment of the included studies	Standard-dose epinephrine compared to placebo improved ROSC, STHA, and STHD, but it did not affect survival with FNO. High-dose epinephrine compared to standard-dose epinephrine improved ROSC and STHA. Vasopressin compared to standard-dose epinephrine improved STHA but not ROSC, whilst the combination of epinephrine and vasopressin compared with epinephrine alone had no effect on these outcomes. Neither standard-dose epinephrine, high-dose epinephrine, vasopressin, nor a combination of epinephrine and vasopressin improved survival with FNO
Holmberg et al. (2022)	To evaluate the effects of vasopressin and glucocorticoids for the treatment of CA	Database: PubMed, Embase, and Cochrane Central Register of Controlled Trials Period: From inception to 30 September 2021 Language: Not limited Search term: Defined Reference tracking or other sources: Yes	- 3 RCTs - 869 participants	Population: IHCA and OHCA patients Intervention: VSE Comparator: Placebo and epinephrine Outcome: ROSC, STHD, and FNO Study design: Randomized and non-randomized trials	Complete and satisfactory quality assessment of the included studies	The use of vasopressin and glucocorticoids compared to placebo resulted in improved ROSC
Li et al. (2020)	To evaluate the efficacy and safety of corticosteroid therapy in CA patients	Database: MEDLINE, Embase, Cochrane Central Register of Controlled Trials, Chinese National Knowledge Infrastructure, and Chinese Biomedical Literature Database Period: From inception to 31 January 2020 Language: Not mentioned Search term: Defined Reference tracking or other sources: Yes	- 5 RCTs - 551 participants	Population: IHCA and OHCA patients Intervention: VSE Comparator: Placebo and epinephrine Outcome: ROSC, STHD, and FNO Study design: RCTs	Complete and satisfactory quality assessment of the included studies	The inherent limitations of the studies included in this review prevented us from reaching definitive conclusions as to the efficacy and safety of corticosteroid therapy in CA patients
Lin et al. (2014)	To review the efficacy of epinephrine in adult OHCA	Database: MEDLINE, Embase, and EBM Reviews (Cochrane Central Register of Controlled Trials, Cochrane Database of Systematic Reviews, Database of Abstracts of Reviews of Effects, Cochrane Method-ology Register, and Health Technology Assessment) Period: From inception to 1 July 2013 Language: Not limited Search term: Defined Reference tracking or other sources: Yes	- 14 RCTs - 12,246 participants	Population: OHCA patients Intervention: Standard-dose epinephrine Comparator: Placebo; high-dose epinephrine; vasopressin; vasopressin and epinephrine Outcome: ROSC, STHA, STHD, and FNO Study design: RCTs	Complete and satisfactory quality assessment of the included studies	There was no benefit of standard-dose epinephrine over placebo, high-dose epinephrine, epinephrine and vasopressin combination, or vasopressin alone, in STHD or FNO. There were improved rates of STHA and ROSC with high-dose epinephrine over standard-dose epinephrine and with standard-dose epinephrine over placebo
Mentzelopoulos et al. (2012)	To determine the possible benefit of vasopressin regarding sustained ROSC, long-term survival, and FNO	Database: PubMed, Embase, and Cochrane Central Register of Controlled Trials Period: From inception to June 2010 Language: English Search term: Defined Reference tracking or other sources: No	- 6 RCTs - 4,745 participants	Population: IHCA and OHCA patients Intervention: Vasopressin and epinephrine with or without steroids Comparator: Placebo and epinephrine Outcome: ROSC, STHD, and FNO Study design: RCTs	Complete and satisfactory quality assessment of the included studies	Vasopressin use in the resuscitation of CA patients was not associated with any overall benefit or harm. However, vasopressin may improve the long-term survival of asystolic patients, especially when average time from collapse to drug administration was < 20 min
Saghafi et al. (2022)	To review the efficacy of combination therapy with VSE in CA and investigate whether this combination therapy improves survival in victims of both IHCA and OHCA	Database: MEDLINE, Scopus, Cochrane Central Register of Controlled Trials, and Google Scholar Period: From inception to October 2021 Language: Not mentioned Search term: Defined Reference tracking or other sources: No	- 3 RCTs - 869 participants	Population: IHCA and OHCA patients Intervention: VSE Comparator: Placebo and epinephrine Outcome: ROSC, STHD, MAP during CPR, MAP 15–20 min after CPR, ventilator free days, renal failure free days, coagulation failure free days, and insulin requirement Study design: RCTs	Complete and satisfactory quality assessment of the included studies	VSE combination therapy may have beneficial effects in terms of the ROSC, renal and circulatory failure free days, and MAP
Satti et al. (2022)	To assess the effect of VSE combination therapy on ROSC after IHCA, and test the conclusiveness of evidence using trial sequential analysis	Database: PubMed, Scopus, and EMBASE Period: From inception to October 2021 Language: English Search term: Defined Reference tracking or other sources: Yes	- 3 RCTs - 869 participants	Population: IHCA patients Intervention: VSE Comparator: Placebo and epinephrine Outcome: ROSC, STHD, and FNO Study design: RCTs	Complete and satisfactory quality assessment of the included studies	This meta-analysis of RCTs demonstrated conclusively that VSE led to improved rates of ROSC
Shah & Mitra (2021)	To evaluate the impact of intraarrest corticosteroids on mortality and FNO in CA patients	Database: Cochrane Central Register of Controlled Trials, Embase, and MEDLINE Period: From inception to 2021 Language: English Search term: Defined Reference tracking or other sources: No	- 5 RCTs, 1 prospective cohort study, 1 retrospective cohort study - 869 participants	Population: IHCA and OHCA patients Intervention: VSE Comparator: Placebo and epinephrine Outcome: ROSC, STHD, and FNO Study design: RCTs and comparative observational studies	Complete and satisfactory quality assessment of the included studies	Corticosteroids given as part of a VSE regimen in CA patients resulted in improved FNO, STHD, and surrogate outcomes that included ROSC and hemodynamics. We found no benefit in CA patients receiving corticosteroids only
Sillberg et al. (2008)	To compare the efficacy of vasopressin and epinephrine used together versus repeated doses of epinephrine alone in CA	Database: MEDLINE, Embase, and Cochrane Central Register of Controlled Trials Period: From inception to 2007 Language: Not limited Search term: Defined Reference tracking or other sources: Yes	- 3 RCTs - 1,226 participants	Population: IHCA and OHCA patients Intervention: Vasopressin and epinephrine Comparator: Epinephrine Outcome: ROSC, STHA, and STHD Study design: RCTs	Appropriate quality assessment of the included studies without the assessment of selective reporting	This review of the combination of vasopressin and epinephrine found trends towards better ROSC but equivocal effects on survival
Zhang et al. (2017)	To compare the efficacy of the combination of vasopressin and epinephrine to epinephrine alone in OHCA patients	Database: PubMed, Embase, Cochrane Library, and Wanfang Period: From inception to February 2017 Language: English and Chinese Search term: Defined Reference tracking or other sources: Yes	- 9 RCTs - 5,047 participants	Population: OHCA patients Intervention: Vasopressin and epinephrine Comparator: Epinephrine Outcome: ROSC Study design: RCTs	Complete and satisfactory quality assessment of the included studies	Combination of vasopressin and epinephrine could improve the ROSC rate of patients from Asia, but patients from other regions could not benefit from it

Abbreviations: CA, cardiac arrest; CPR, cardiopulmonary resuscitation; FNO, favorable neurological outcomes; IHCA, in-hospital cardiac arrest; MAP, mean arterial pressure; OHCA, out-of-hospital cardiac arrest; PICOS, population, intervention, comparator, outcome, and study design; RCT, randomized controlled trial; RoB, risk of bias; ROSC, return of spontaneous circulation; STHA, survival to hospital admission; STHD, survival to hospital discharge; VSE, vasopressin, steroids, and epinephrine

**Table 3:** Original studies reviewed in each systematic review

		Original Study															
		Andersen et al. (2021)	Callaway et al. (2006)	Ducros et al. (2011)	Gueugniaud et al. (2008)	He et al. (2010)	Hu, & Ma (2008)	Li et al. (1999)	Lindner et al. (1997)	Mentzelopoulos et al. (2009)	Mentzelopoulos et al. (2013)	Mukoyama et al. (2009)	Ong et al. (2012)	Stiell et al. (2001)	Wenzel et al. (2004)	Xiao et al. (2007)	Yang (2012)
		Denmark	United States	France	France	China	China	China	Germany	Greece	Greece	Japan	Singapore	Canada	Austria, Germany, & Switzerland	China	China
		N = 501	N = 325	N = 44	N = 2,894	N = 102	N = 78	N = 83	N = 40	N = 100	N = 268	N = 336	N = 727	N = 200	N = 1,186	N = 69	N = 90
Systematic Review	Abdelazeem et al. (2022)	✓								✓	✓						
	Belletti et al. (2018)		✓	✓	✓				✓	✓	✓	✓	✓	✓	✓		
	Finn et al. (2019)		✓	✓	✓			✓	✓			✓	✓	✓	✓		
	Holmberg et al. (2022)	✓								✓	✓						
	Li et al. (2020)									✓	✓						
	Lin et al. (2014)		✓	✓	✓				✓			✓	✓		✓		
	Mentzelopoulos et al. (2012)		✓		✓				✓	✓				✓	✓		
	Saghafi et al. (2022)	✓								✓	✓						
	Satti et al. (2022)	✓								✓	✓						
	Shah & Mitra (2021)									✓	✓						
	Sillberg et al. (2008)		✓											✓	✓		
	Zhang et al. (2017)		✓	✓	✓	✓	✓						✓		✓	✓	✓

### Outcome Evaluation

Table 4 presents the results of the pairwise meta-analyses performed in the included SRs.

**Table 4:** Results of the pairwise meta-analyses performed in the included systematic reviews

Outcome	Citation	Number of Studies		IHCA	OHCA	Intervention Group		Control Group		Result			
		RCT	Non-RCT			Treatment	Number of Participants	Treatment	Number of Participants	Effect	p-value	I^2^	Sensitivity Analysis
ROSC	Abdelazeem et al. (2022)	3	0	✓		VSE	415	Placebo + Epinephrine	454	RR = 1.32 (1.18, 1.47)	< 0.00001	0%	Similar result
	Finn et al. (2019)	6	0	✓	✓	Vasopressin	1,285	Epinephrine	1,246	RR = 1.10 (0.90, 1.33)	0.36	61%	
		2	0	✓		Vasopressin	124	Epinephrine	118	RR = 1.76 (0.40, 7.71)	0.45	84%	
		3	0		✓	Vasopressin	787	Epinephrine	775	RR = 1.05 (0.80, 1.39)	0.72	56%	
		3	0		✓	Vasopressin + Epinephrine	1,623	Epinephrine	1,626	RR = 0.97 (0.87, 1.08)	0.57	0%	
	Holmberg et al. (2022)	3	0	✓		VSE	415	Placebo + Epinephrine	454	OR = 2.09 (1.54, 2.84)	< 0.05*	63%	
	Lin et al. (2014)	6	0		✓	Epinephrine	2,596	Vasopressin + Epinephrine	2,606	RR = 0.96 (0.89, 1.04)	0.31	0%	
	Mentzelopoulos et al. (2012)	6	0	✓	✓	Vasopressin + Epinephrine ± Steroids	2,370	Placebo + Epinephrine	2,375	OR = 1.25 (0.90, 1.74)	0.18	71%	Similar result
	Saghafi et al. (2022)	3	0	✓		VSE	415	Placebo + Epinephrine	454	OR = 2.28 (1.30, 3.99)	0.004*	63%	Similar result
	Satti et al. (2022)	3	0	✓		VSE	415	Placebo + Epinephrine	454	RR = 1.41 (1.25, 1.59)	< 0.00001	0%	
	Shah & Mitra (2021)	2	0	✓		VSE	178	Placebo + Epinephrine	190	RR = 1.35 (1.12, 1.64)	0.002*	37%	
	Zhang et al. (2017)	9	0		✓	Vasopressin + Epinephrine	2,535	Epinephrine	2,512	OR = 1.67 (1.13, 2.49)	0.01*	83%	
STHA	Finn et al. (2019)	3	0	✓	✓	Vasopressin	983	Epinephrine	970	RR = 1.27 (1.04, 1.54)	0.018*	27%	
		3	0		✓	Vasopressin + Epinephrine	1,623	Epinephrine	1,626	RR = 0.95 (0.83, 1.08)	0.40	0%	
	Lin et al. (2014)	5	0		✓	Epinephrine	2,438	Vasopressin + Epinephrine	2,439	RR = 0.88 (0.73, 1.06)	0.17	56%	
STHD	Abdelazeem et al. (2022)	3	0	✓		VSE	415	Placebo + Epinephrine	454	RR = 1.76 (0.68, 4.56)	0.25	76%	Favours VSE
		2	0	✓		VSE	178	Placebo + Epinephrine	190	RR = 2.58 (1.36, 4.91)	0.004*	0%	
	Finn et al. (2019)	6	0	✓	✓	Vasopressin	1,274	Epinephrine	1,237	RR = 1.25 (0.84, 1.85)	0.27	29%	
		2	0	✓		Vasopressin	124	Epinephrine	118	RR = 2.21 (0.29, 17.06)	0.45	77%	
		3	0		✓	Vasopressin	776	Epinephrine	766	RR = 1.26 (0.76, 2.07)	0.37	29%	
		3	0		✓	Vasopressin + Epinephrine	1,620	Epinephrine	1,622	RR = 0.76 (0.47, 1.22)	0.25	0%	
	Holmberg et al. (2022)	3	0	✓		VSE	415	Placebo + Epinephrine	454	OR = 1.39 (0.90, 2.14)	> 0.05	73%	
	Lin et al. (2014)	5	0		✓	Epinephrine	2,438	Vasopressin + Epinephrine	2,439	RR = 1.00 (0.69, 1.44)	0.99	25%	
	Mentzelopoulos et al. (2012)	6	0	✓	✓	Vasopressin + Epinephrine ± Steroids	2,356	Placebo + Epinephrine	2,362	OR = 1.13 (0.71, 1.78)	0.61	46%	Similar result
	Saghafi et al. (2022)	3	0	✓		VSE	415	Placebo + Epinephrine	454	OR = 2.08 (0.64, 6.80)	0.225	79%	Similar result
	Shah & Mitra (2021)	2	0	✓		VSE	178	Placebo + Epinephrine	190	RR = 2.58 (1.36, 4.91)	0.004*	0%	
FNO	Abdelazeem et al. (2022)	3	0	✓		VSE	415	Placebo + Epinephrine	454	RR = 1.80 (0.81, 4.01)	0.15	56%	Favours VSE
		2	0	✓		VSE	178	Placebo + Epinephrine	190	RR = 2.84 (1.36, 5.94)	0.006*	0%	
	Finn et al. (2019)	4	0	✓	✓	Vasopressin	1,223	Epinephrine	1,183	RR = 0.82 (0.54, 1.25)	0.36	0%	
	Holmberg et al. (2022)	3	0	✓		VSE	415	Placebo + Epinephrine	454	OR = 1.64 (0.99, 2.72)	> 0.05	63%	
	Lin et al. (2014)	3	0		✓	Epinephrine	2,402	Vasopressin + Epinephrine	2,405	RR = 1.32 (0.88, 1.98)	0.18	0%	
	Mentzelopoulos et al. (2012)	4	0	✓	✓	Vasopressin + Epinephrine ± Steroids	2,158	Placebo + Epinephrine	2,172	OR = 0.87 (0.49, 1.52)	0.62	46%	Similar result
	Shah & Mitra (2021)	2	0	✓		VSE	178	Placebo + Epinephrine	190	RR = 2.84 (1.36, 5.94)	0.006*	0%	
MAP during CPR	Saghafi et al. (2022)	2	0	✓		VSE	178	Placebo + Epinephrine	190	SMD = 1.07 (0.85, 1.29)	< 0.001*	0%	
MAP 15–20 min after CPR	Saghafi et al. (2022)	2	0	✓		VSE	178	Placebo + Epinephrine	190	SMD = 0.83 (0.55, 1.11)	< 0.001*	0%	
Ventilator free days	Saghafi et al. (2022)	3	0	✓		VSE	415	Placebo + Epinephrine	454	SMD = 0.20 (-0.68, 1.08)	0.838	95%	
Renal failure free days	Saghafi et al. (2022)	2	0	✓		VSE	178	Placebo + Epinephrine	190	SMD = 0.59 (0.31, 0.87)	< 0.001*	0%	
Coagulation failure free days	Saghafi et al. (2022)	2	0	✓		VSE	178	Placebo + Epinephrine	190	SMD = 0.40 (0.13, 0.68)	0.004*	0%	
Insulin requirement	Saghafi et al. (2022)	3	0	✓		VSE	415	Placebo + Epinephrine	454	OR = 1.71 (1.32, 2.21)	< 0.001*	0%	

^*^ Statistically significant

Abbreviations: CPR, cardiopulmonary resuscitation; FNO, favorable neurological outcomes; IHCA, in-hospital cardiac arrest; MAP, mean arterial pressure; OHCA, out-of-hospital cardiac arrest; OR, odds ratio; RCT, randomized controlled trial; ROSC, return of spontaneous circulation; RR, risk ratio; SMD, standardized mean difference; STHA, survival to hospital admission; STHD, survival to hospital discharge; VSE, vasopressin, steroids, and epinephrine

#### Return of Spontaneous Circulation

##### Vasopressin Alone Versus Epinephrine Alone

Finn et al. [13] performed three pairwise meta-analyses to compare vasopressin alone and epinephrine alone in terms of their effects on ROSC. They discovered no significant difference in ROSC among patients with IHCA (risk ratio [RR], 1.76; 95% CI, 0.40–7.71; *p* = 0.45), those with OHCA (RR, 1.05; 95% CI, 0.80–1.39; *p* = 0.72), and those with IHCA or OHCA (RR, 1.10; 95% CI, 0.90–1.33; *p* = 0.36).

##### Vasopressin–Epinephrine Versus Epinephrine Alone

Three pairwise meta-analyses were performed to compare vasopressin–epinephrine and epinephrine alone in terms of their effects on ROSC [13, 34, 38]. Zhang et al. [38] reported a significant increase in ROSC in patients with OHCA receiving vasopressin–epinephrine compared with that in those receiving epinephrine alone (OR, 1.67; 95% CI, 1.13–2.49; *p* = 0.01). By contrast, no significant difference was observed in ROSC among patients with OHCA in the meta-analyses conducted by Finn et al. [13] (RR, 0.97; 95% CI, 0.87–1.08; *p* = 0.57) and Lin et al. [34] (RR, 0.96; 95% CI, 0.89–1.04; *p* = 0.31). Sillberg et al. [37] narratively described the results of two RCTs [41, 53], which were included in the aforementioned meta-analyses.

**Fig. 2:**
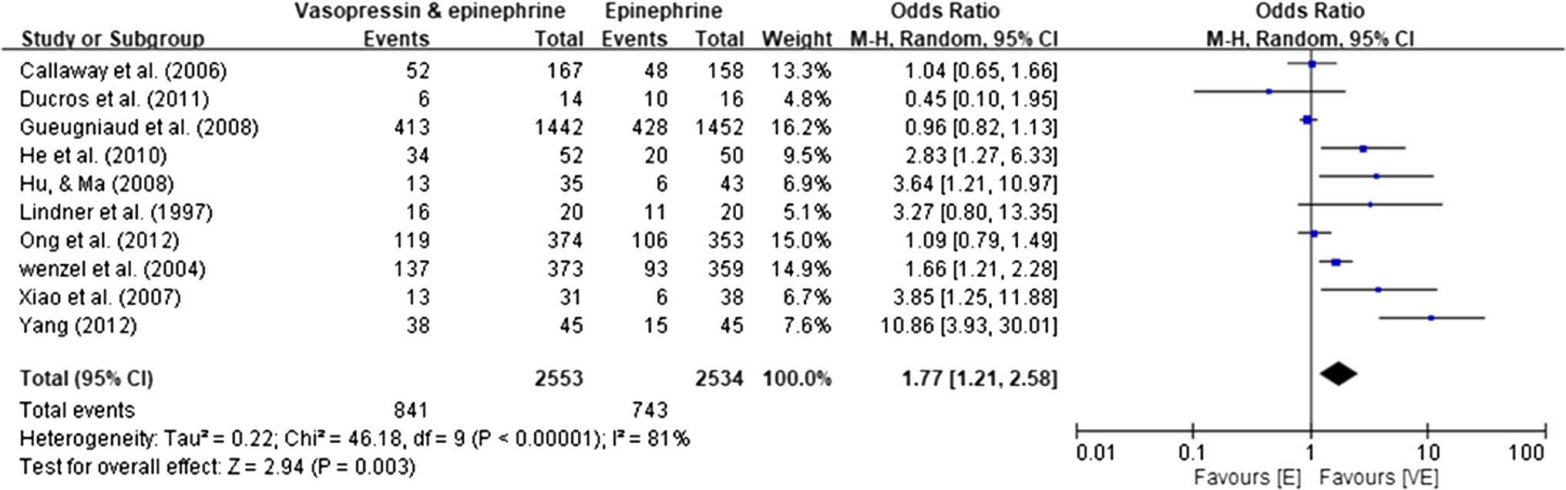
Forest Plot for the Comparison of Vasopressin-Epinephrine and Epinephrine Alone on the Return of Spontaneous Circulation

No pairwise meta-analysis included all RCTs. Three RCTs [41–43] were common among the meta-analyses performed by Finn et al. [13], Lin et al. [34], and Zhang et al. [38]. Furthermore, two RCTs [51, 53] were common between the meta-analyses performed by Lin et al. [34] and Zhang et al. [38]. After removing the duplicate RCTs, we performed a re-meta-analysis of 10 RCTs [41–45, 47, 51, 53–55] and discovered a significant increase in ROSC in patients with OHCA receiving vasopressin–epinephrine compared with that in those receiving epinephrine alone (OR, 1.77; 95% CI, 1.21–2.58; *p* = 0.003); however, high statistical heterogeneity (*I*^2^ = 81%) was noted (Fig. 2). A sensitivity analysis was performed by removing the included RCTs one by one to assess the robustness of the results; the results indicated no significant change in statistical heterogeneity (*I*^2^ = 71%–83%).

##### Vasopressin–Steroid–Epinephrine Versus Epinephrine–Placebo

Six pairwise meta-analyses were conducted to compare VSE and epinephrine–placebo in terms of their effects on ROSC [9, 16, 17, 28, 35, 39]. A significant increase in ROSC was observed in patients with IHCA receiving VSE compared with that in those receiving epinephrine–placebo in the meta-analyses conducted by Abdelazeem et al. [16] (RR, 1.32; 95% CI, 1.18–1.47; *p* < 0.00001), Holmberg et al. [28] (OR, 2.09; 95% CI, 1.54–2.84; *p* < 0.05), Saghafi et al. [9] (OR, 2.28; 95% CI, 1.30–3.99; *p* = 0.004), Satti et al. [39] (RR, 1.41; 95% CI, 1.25–1.59; *p* < 0.00001), and Shah and Mitra [17] (RR, 1.35; 95% CI, 1.12–1.64; *p* = 0.002). By contrast, Mentzelopoulos et al. [35] found no significant increase in ROSC in patients with IHCA or OHCA receiving vasopressin and epinephrine with or without steroids compared with that in those receiving epinephrine–placebo (OR, 1.25; 95% CI, 0.90–1.74; *p* = 0.18). Similar results were obtained in the sensitivity analyses performed by Abdelazeem et al. [16], Saghafi et al. [9], and Mentzelopoulos et al. [35]. Li et al. [33] narratively described the results of two RCTs [48, 49], which were included in the aforementioned meta-analyses.

##### Results of Network Meta-Analysis

In their network meta-analysis involving patients with IHCA or OHCA, Belletti et al. [26] found a significant increase in ROSC in patients receiving VSE compared with that in those receiving epinephrine or other drugs.

#### Survival to Hospital Admission

##### Vasopressin Alone Versus Epinephrine Alone

Finn et al. [13] performed a pairwise meta-analysis of vasopressin alone and epinephrine alone in terms of their effects on STHA. They discovered a significant increase in STHA in patients with IHCA or OHCA receiving vasopressin alone compared with that in those receiving epinephrine alone (RR, 1.27; 95% CI, 1.04–1.54; *p* = 0.018).

##### Vasopressin–Epinephrine Versus Epinephrine Alone

Two pairwise meta-analyses were performed to compare vasopressin–epinephrine and epinephrine alone in terms of their effects on STHA [13, 34]. No significant difference was found in STHA among patients with OHCA in the meta-analyses conducted by Finn et al. [13] (RR, 0.95; 95% CI, 0.83–1.08; *p* = 0.40) and Lin et al. [34] (RR, 0.88; 95% CI, 0.73–1.06; *p* = 0.17). Sillberg et al. [37] narratively described the results of two RCTs [41, 53], which were included in the aforementioned meta-analyses.

#### Survival to Hospital Discharge

##### Vasopressin Alone Versus Epinephrine Alone

Finn et al. [13] performed three pairwise meta-analyses to compare vasopressin alone and epinephrine alone in terms of their effects on STHD. They discovered no significant difference in STHD among patients with IHCA (RR, 2.21; 95% CI, 0.29–17.06; *p* = 0.45), those with OHCA (RR, 1.26; 95% CI, 0.76–2.07; *p* = 0.37), and those with IHCA or OHCA (RR, 1.25; 95% CI, 0.84–1.85; *p* = 0.27).

##### Vasopressin–Epinephrine Versus Epinephrine Alone

Two pairwise meta-analyses were conducted to compare vasopressin–epinephrine and epinephrine alone in terms of their effects on STHD [13, 34]. No significant difference was observed in STHD among patients with OHCA in the meta-analyses conducted by Finn et al. [13] (RR, 0.76; 95% CI, 0.47–1.22; *p* = 0.25) and Lin et al. [34] (RR, 1.00; 95% CI, 0.69–1.44; *p* = 0.99). Sillberg et al. [37] narratively described the result of one RCT [53], which was included in the aforementioned meta-analyses.

##### Vasopressin–Steroid–Epinephrine Versus Epinephrine–Placebo

Six pairwise meta-analyses were performed to compare VSE and epinephrine–placebo in terms of their effects on STHD [9, 16, 17, 28, 35]. No significant difference was observed in STHD among patients with IHCA in the meta-analyses conducted by Abdelazeem et al. [16] (RR, 1.76; 95% CI, 0.68–4.56; *p* = 0.25), Holmberg et al. [28] (OR, 1.39; 95% CI, 0.90–2.14; *p* > 0.05), and Saghafi et al. [9] (OR, 2.08; 95% CI, 0.64–6.80). Three RCTs [40, 48, 49] were common among the aforementioned three meta-analyses. Abdelazeem et al. [16] performed a sensitivity analysis after removing the study of Andersen et al. [40] and found a significant increase in STHD in patients with IHCA receiving VSE compared with that in those receiving epinephrine–placebo (RR, 2.58; 95% CI, 1.36–4.91; *p* = 0.004). The same result was reported in the meta-analysis performed by Shah and Mitra [17], which included two RCTs [48, 49] that were also included in the aforementioned meta-analysis following the sensitivity analysis. By contrast, Mentzelopoulos et al. [35] discovered no significant increase in STHD in patients with IHCA or OHCA receiving vasopressin and epinephrine with or without steroids compared with that in those receiving epinephrine–placebo (OR, 1.13; 95% CI, 0.71–1.78; *p* = 0.61). The sensitivity analysis performed in the aforementioned SR revealed similar results [35]. Li et al. [33] and Satti et al. [39] narratively described the results of the RCTs [40, 48, 49], which were included in the aforementioned meta-analyses.

##### Results of Network Meta-Analysis

In their network meta-analysis involving patients with IHCA or OHCA, Belletti et al. [26] found a significant increase in survival at the longest follow-up available in patients receiving VSE compared with that in those receiving epinephrine or other drugs. This increase was particularly notable for patients with IHCA.

#### Favorable Neurological Outcomes

##### Vasopressin Alone Versus Epinephrine Alone

Finn et al. [13] performed a pairwise meta-analysis to compare vasopressin alone and epinephrine alone in terms of their effects on FNO. They discovered no significant difference in FNO among patients with IHCA or OHCA (RR, 0.82; 95% CI, 0.54–1.25; *p* = 0.36).

##### Vasopressin–Epinephrine Versus Epinephrine Alone

Lin et al. [34] performed a pairwise meta-analysis to compare vasopressin–epinephrine and epinephrine alone in terms of their effects on FNO. They discovered no significant difference in FNO among patients with OHCA (RR, 1.32; 95% CI, 0.88–1.98; *p* = 0.18).

##### Vasopressin–Steroid–Epinephrine Versus Epinephrine–Placebo

Five pairwise meta-analyses were conducted to compare VSE and epinephrine–placebo in terms of their effects on FNO [16, 17, 28, 35]. No significant difference was observed in FNO among patients with IHCA in the meta-analyses conducted by Abdelazeem et al. [16] (RR, 1.80; 95% CI, 0.81–4.01; *p* = 0.15) and Holmberg et al. [28] (OR, 1.64; 95% CI, 0.99–2.72; *p* > 0.05). Three RCTs [40, 48, 49] were common among the aforementioned two meta-analyses. Abdelazeem et al. [16] performed a sensitivity analysis after removing the study of Andersen et al. [40] and found a significant increase in FNO in patients with IHCA receiving VSE compared with that in those receiving epinephrine–placebo (RR, 2.84; 95% CI, 1.36–5.94; *p* = 0.006). Shah and Mitra [17] reported the same result in their meta-analysis conducted using two RCTs [48, 49] that were also included in the aforementioned meta-analysis following the sensitivity analysis. By contrast, Mentzelopoulos et al. [35] found no significant increase in FNO in patients with IHCA or OHCA receiving vasopressin and epinephrine with or without steroids compared with that in those receiving epinephrine–placebo (OR, 0.87; 95% CI, 0.49–1.52; *p* = 0.62). Li et al. [33] and Satti et al. [39] narratively described the results of the RCTs [40, 48, 49], which were included in the aforementioned meta-analyses.

##### Results of Network Meta-Analysis

In their network meta-analysis involving patients with IHCA or OHCA, Belletti et al. [26] found a significant increase in FNO in patients receiving VSE compared with that in those receiving epinephrine or other drugs.

#### Other Outcomes

##### Vasopressin–Steroid–Epinephrine Versus Epinephrine–Placebo

Saghafi et al. [9] discovered significant increases in the following parameters for patients with IHCA receiving VSE compared with the increases for those receiving epinephrine–placebo: (1) MAP during CPR (standardized mean difference [SMD], 1.07 mmHg; 95% CI, 0.85–1.29 mmHg; *p* < 0.001), (2) MAP 15–20 min after CPR (SMD, 0.83 mmHg; 95% CI, 0.55–1.11 mmHg; *p* < 0.001), (3) renal failure–free days (SMD, 0.59 day; 95% CI, 0.31–0.87 day; *p* < 0.001), (4) coagulation failure–free days (SMD, 0.40 day; 95% CI, 0.13–0.68 day; *p* = 0.004), and (5) insulin requirement (OR, 1.71; 95% CI, 1.32–2.21; *p* < 0.001). By contrast, no significant difference was observed in ventilator–free days among patients with IHCA (SMD, 0.20 day; 95% CI, − 0.68 to 1.08 day; *p* = 0.838) [9].

## Discussion

### Principal Findings

Finn et al. [13] found no significant difference in ROSC, STHD, and FNO but a significant increase in STHA in patients with IHCA or OHCA receiving vasopressin alone compared with the corresponding findings in those receiving epinephrine alone. These findings indicate that vasopressin and epinephrine exhibit similar effectiveness in achieving ROSC and maintaining STHD and FNO; however, vasopressin alone is more effective in maintaining STHA in patients with IHCA or OHCA than is epinephrine alone [13]. This difference might have occurred because vasopressin does not increase myocardial oxygen consumption, which leads to myocardial dysfunction, and thus preserves cardiac function to maintain STHA [12].

Finn et al. [13] and Lin et al. [34] found no significant difference in ROSC, STHA, STHD, and FNO between patients with OHCA receiving vasopressin–epinephrine and those receiving epinephrine alone. However, the meta-analysis performed by Zhang et al. [38] using 9 of 10 RCTs revealed that patients with OHCA receiving vasopressin–epinephrine exhibited a significant increase in ROSC. The finding is further supported by our re-meta-analysis performed using all 10 RCTs. This finding indicates that vasopressin–epinephrine is more effective in achieving ROSC than is epinephrine alone and that both types of drug therapy have similar effectiveness in maintaining STHA, STHD, and FNO in patients with OHCA. The aforementioned difference might have occurred because vasopressin and epinephrine improve coronary perfusion pressure through distinct mechanisms, thus exerting a synergistic effect to achieve ROSC [56].

Although vasopressin-epinephrine is more effective in achieving ROSC in patients with OHCA and vasopressin alone is more effective in maintaining STHA in patients with IHCA or OHCA compared with epinephrine alone, neither of them is more effective in maintaining STHD and FNO. These findings may be explained by the side effects of vasopressin: (1) vasopressin causes coronary vasoconstriction with decreased coronary blood flow and weaker cardiac contractility [57, 58], (2) vasopressin leads to systemic vasoconstriction with increased cardiac afterload and higher risk of cardiac pathology [58–60], and (3) vasopressin may participate in cardiac inflammation and fibrosis by promoting IL-1β expression through the β-arrestin2-mediated NF-κB signaling pathway in humans [61]. The subsequent activation of the apelin system, which is opposed to the vasopressin system, may be another explanation. The administration of vasopressin increases plasma osmolality and subsequently activates the apelin system, which causes systemic vasodilation and decreased blood pressure, thus reducing coronary perfusion pressure and coronary blood flow [62–64].

In five SRs, a significant increase was noted in ROSC in patients with IHCA receiving VSE compared with that in those receiving epinephrine–placebo [9, 16, 17, 28, 39]. The meta-analysis results of these five SRs were based on two RCTs administering VSE during CPR and hydrocortisone for 7 days [48, 49] with or without the RCT administering VSE during CPR only [40]. Abdelazeem et al. [16] and Shah and Mitra [17] discovered a significant increase in STHD and FNO in patients with IHCA receiving VSE. The sensitivity analysis of Abdelazeem et al. [16] proved a significant increase in STHD and FNO in patients receiving VSE during CPR and hydrocortisone for 7 days [48, 49] but not in patients receiving VSE during CPR only [40]. Furthermore, Belletti et al. [26] revealed a significant increase in survival at the longest follow-up available in patients with ICHA receiving VSE compared with that in those receiving epinephrine or other drugs. These findings indicate that VSE is the most effective drug combination for achieving ROSC and maintaining survival and FNO in patients with IHCA. The aforementioned findings may be explained by the following reasons: (1) vasopressin and epinephrine exert a synergistic effect to improve coronary perfusion pressure [56], (2) steroids augment vascular responsiveness to vasopressors, thus enhancing vascular tone and optimizing hemodynamic stability [65], and (3) steroids reduce oxidative stress and systemic inflammatory response after CA, thus ameliorating myocardial apoptosis and cerebral injury [66].

Saghafi et al. [9] found significant differences in MAP during and after CPR, renal failure–free days, coagulation failure–free days, and insulin requirement but not in ventilator–free days between patients with IHCA receiving VSE and those receiving epinephrine–placebo. Even when ROSC is achieved, various degrees of ischemia and damage may occur in all tissues and organs, increasing the risk of multiple organ failure [67]. It is essential to improve coronary perfusion and cardiac contractility and maintain microcirculation to minimize the incidence of multiple organ failure [68]. Since MAP was increased during and after CPR in patients with IHCA receiving VSE, the incidence of multiple organ failure was minimized by improving coronary perfusion and cardiac contractility and maintaining microcirculation. Accordingly, increases in renal failure–free days and coagulation failure–free days were observed [9]. Hyperglycemia is an adverse effect of steroids and is managed with insulin [69]. This may explain why an SR reported a greater requirement for insulin in patients with IHCA receiving VSE [9].

### Strengths and Limitations

This review has some strengths. We extensively searched five major academic databases to identify all relevant SRs for this review. Moreover, two reviewers independently functioned at each stage of this review to ensure the eligibility and quality of the included SRs and the validity of the data extracted from the SRs.

Our study has some limitations. Although we performed a comprehensive search for all relevant SRs, some potentially eligible SRs, such as those published in a language other than English, might have been missed. Furthermore, high levels of heterogeneity were observed in the results of some meta-analyses. This heterogeneity might be attributed to the wide variation in the practice of basic and advanced life support medicine because the time interval between the first and last RCTs included in the meta-analyses was more than two decades. Another reason may be the differences in the etiology of CA, the quality of CPR, the provision of ancillary care, and the advancement of post-CA treatment. Because of the high heterogeneity, the results of the meta-analyses should be interpreted cautiously.

### Implications for Future Research and Practice

This review has some implications for future research and practice. Although VSE was demonstrated to be the most effective drug combination for improving CA outcomes, the results of the meta-analyses were based on only two RCTs involving patients with IHCA [48, 49]. In in-hospital settings, health-care staff are well trained in managing CA, and equipment is readily available for providing advanced life support and post-CA treatment, thus favoring the aforementioned findings [26]. Therefore, future studies must evaluate the effectiveness of VSE in patients with OHCA. Moreover, in approximately 40% of the patients included in the two RCTs, CA occurred due to hypotension or respiratory failure [48, 49]. A considerable proportion of these patients might have had septic shock, chest infection, or acutely exacerbated asthma or chronic obstructive pulmonary disease, for which steroids might have been beneficial [17]. Accordingly, future studies should investigate the effectiveness of VSE by including a subgroup analysis by CA cause. In addition, the standard dosages of vasopressin and epinephrine for cardiac resuscitation have been established, but the types and standard dosages of steroids have not been established yet. There is a need to verify the optimal prescription of steroids to maximize the effectiveness of VSE. Hence, future studies should determine the types and standard dosages of steroids for cardiac resuscitation. No study has focused on the combination of vasopressin and steroids (vasopressin-steroid). Because epinephrine increases myocardial oxygen consumption and leads to myocardial dysfunction, removing epinephrine from VSE may further enhance its effectiveness. Therefore, future studies should evaluate the effectiveness of vasopressin–steroid in improving the CA outcomes.

Currently, the American Heart Association and the European Resuscitation Council do not recommend vasopressin and steroids for treating CA [5, 6]. The findings of this review support not using vasopressin alone or vasopressin–epinephrine because the long-term outcomes of CA do not improve regardless of whether vasopressin is administrated as an alternative or an adjunct to epinephrine. On the other hand, this review synthesised the best available evidence and found that VSE is the most effective drug combination for improving the short- and long-term outcomes of IHCA. Therefore, VSE is recommended to be used in patients with IHCA, particularly in those patients whose etiologies are related to inflammation. Moreover, the findings of this review indicate that administering VSE during CPR is effective in achieving ROSC, and continuing steroids for 7 days is essential to maintain STHD and FNO. Accordingly, steroids should be continued for at least a week after administering VSE during CPR. If there is additional evidence in the future, VSE may be used to improve the short- and long-term outcomes of OHCA and CA of various etiologies.

## Conclusions

The findings of this review indicate that VSE is the most effective drug combination for improving the short- and long-term outcomes of IHCA. Therefore, it is recommended to use VSE in patients with IHCA, especially when the etiologies are related to inflammation. Additionally, steroids should be continued for at least a week after administering VSE during CPR. Future studies should investigate the effectiveness of VSE in patients with OHCA and those with CA of various etiologies, the types and standard dosages of steroids for cardiac resuscitation, and the effectiveness of vasopressin–steroid in improving CA outcomes.

## Supplementary Information

Below is the link to the electronic supplementary material.

Supplementary file1 (DOCX 35 KB)

## Data Availability

The data and material underlying this article are available in the article and in its online supplementary material.
