# Supplementary material for: Effectiveness of Vasopressin Against Cardiac Arrest: A Systematic Review of Systematic Reviews
Source: Cardiovasc Drugs Ther. 2024 Mar 12;39(5):1163–83. doi: 10.1007/s10557-024-07571-3 (PMC12602646; doi:10.1007/s10557-024-07571-3)
Supplement: Supplementary file 1 — Supplementary file1 (DOCX 35 KB) [file 10557_2024_7571_MOESM1_ESM.docx]

**Supplementary Information 1**

**PRISMA 2020 Checklist**

| **Section and Topic** | **Item #** | **Checklist item** | **Location where item is reported** |
| --- | --- | --- | --- |
| **TITLE** | | |  |
| Title | 1 | Identify the report as a systematic review. | P.3 |
| **ABSTRACT** | | |  |
| Abstract | 2 | See the PRISMA 2020 for Abstracts checklist. | P.3-4 |
| **INTRODUCTION** | | |  |
| Rationale | 3 | Describe the rationale for the review in the context of existing knowledge. | P.5-7 |
| Objectives | 4 | Provide an explicit statement of the objective(s) or question(s) the review addresses. | P.7 |
| **METHODS** | | |  |
| Eligibility criteria | 5 | Specify the inclusion and exclusion criteria for the review and how studies were grouped for the syntheses. | P.7-8 |
| Information sources | 6 | Specify all databases, registers, websites, organisations, reference lists and other sources searched or consulted to identify studies. Specify the date when each source was last searched or consulted. | P.8-9 & 11 |
| Search strategy | 7 | Present the full search strategies for all databases, registers and websites, including any filters and limits used. | P.8-9; Supplementary information 2 |
| Selection process | 8 | Specify the methods used to decide whether a study met the inclusion criteria of the review, including how many reviewers screened each record and each report retrieved, whether they worked independently, and if applicable, details of automation tools used in the process. | P.9 |
| Data collection process | 9 | Specify the methods used to collect data from reports, including how many reviewers collected data from each report, whether they worked independently, any processes for obtaining or confirming data from study investigators, and if applicable, details of automation tools used in the process. | P.10 |
| Data items | 10a | List and define all outcomes for which data were sought. Specify whether all results that were compatible with each outcome domain in each study were sought (e.g. for all measures, time points, analyses), and if not, the methods used to decide which results to collect. | P.10 |
|  | 10b | List and define all other variables for which data were sought (e.g. participant and intervention characteristics, funding sources). Describe any assumptions made about any missing or unclear information. | P.10 |
| Study risk of bias assessment | 11 | Specify the methods used to assess risk of bias in the included studies, including details of the tool(s) used, how many reviewers assessed each study and whether they worked independently, and if applicable, details of automation tools used in the process. | P.9-10; Supplementary information 3 |
| Effect measures | 12 | Specify for each outcome the effect measure(s) (e.g. risk ratio, mean difference) used in the synthesis or presentation of results. | P.10-11 |
| Synthesis methods | 13a | Describe the processes used to decide which studies were eligible for each synthesis (e.g. tabulating the study intervention characteristics and comparing against the planned groups for each synthesis (item #5)). | P. 10-11 |
|  | 13b | Describe any methods required to prepare the data for presentation or synthesis, such as handling of missing summary statistics, or data conversions. | P. 10-11 |
|  | 13c | Describe any methods used to tabulate or visually display results of individual studies and syntheses. | P. 10-11 |
|  | 13d | Describe any methods used to synthesize results and provide a rationale for the choice(s). If meta-analysis was performed, describe the model(s), method(s) to identify the presence and extent of statistical heterogeneity, and software package(s) used. | P. 10-11 |
|  | 13e | Describe any methods used to explore possible causes of heterogeneity among study results (e.g. subgroup analysis, meta-regression). | P. 10-11 |
|  | 13f | Describe any sensitivity analyses conducted to assess robustness of the synthesized results. | P. 10-11 |
| Reporting bias assessment | 14 | Describe any methods used to assess risk of bias due to missing results in a synthesis (arising from reporting biases). | NA |
| Certainty assessment | 15 | Describe any methods used to assess certainty (or confidence) in the body of evidence for an outcome. | NA |
| **RESULTS** | | |  |
| Study selection | 16a | Describe the results of the search and selection process, from the number of records identified in the search to the number of studies included in the review, ideally using a flow diagram. | P.11; Figure 1 |
|  | 16b | Cite studies that might appear to meet the inclusion criteria, but which were excluded, and explain why they were excluded. | P.11; Supplementary information 4 |
| Study characteristics | 17 | Cite each included study and present its characteristics. | P.12; Table 2 |
| Risk of bias in studies | 18 | Present assessments of risk of bias for each included study. | P.11-12; Table 1 |
| Results of individual studies | 19 | For all outcomes, present, for each study: (a) summary statistics for each group (where appropriate) and (b) an effect estimate and its precision (e.g. confidence/credible interval), ideally using structured tables or plots. | P.12-20; Table 4 |
| Results of syntheses | 20a | For each synthesis, briefly summarise the characteristics and risk of bias among contributing studies. | P.12-20; Table 4 |
|  | 20b | Present results of all statistical syntheses conducted. If meta-analysis was done, present for each the summary estimate and its precision (e.g. confidence/credible interval) and measures of statistical heterogeneity. If comparing groups, describe the direction of the effect. | P.12-20; Table 4 |
|  | 20c | Present results of all investigations of possible causes of heterogeneity among study results. | P.12-20; Table 4 |
|  | 20d | Present results of all sensitivity analyses conducted to assess the robustness of the synthesized results. | P.12-20; Table 4 |
| Reporting biases | 21 | Present assessments of risk of bias due to missing results (arising from reporting biases) for each synthesis assessed. | NA |
| Certainty of evidence | 22 | Present assessments of certainty (or confidence) in the body of evidence for each outcome assessed. | NA |
| **DISCUSSION** | | |  |
| Discussion | 23a | Provide a general interpretation of the results in the context of other evidence. | P.20-24 |
|  | 23b | Discuss any limitations of the evidence included in the review. | P.24-25 |
|  | 23c | Discuss any limitations of the review processes used. | P.24-25 |
|  | 23d | Discuss implications of the results for practice, policy, and future research. | P.25-27 |
| **OTHER INFORMATION** | | |  |
| Registration and protocol | 24a | Provide registration information for the review, including register name and registration number, or state that the review was not registered. | P.7 |
|  | 24b | Indicate where the review protocol can be accessed, or state that a protocol was not prepared. | P.7 |
|  | 24c | Describe and explain any amendments to information provided at registration or in the protocol. | P.11 |
| Support | 25 | Describe sources of financial or non-financial support for the review, and the role of the funders or sponsors in the review. | P.27 |
| Competing interests | 26 | Declare any competing interests of review authors. | P.27 |
| Availability of data, code and other materials | 27 | Report which of the following are publicly available and where they can be found: template data collection forms; data extracted from included studies; data used for all analyses; analytic code; any other materials used in the review. | N/A |

**Supplementary Information 2**

**Search strategy (MEDLINE)**

#1 exp Vasopressins/

#2 (vasopressin* or vasopressor* or antidiuretic hormone* or argipressin or pitressin).mp. [mp=title, abstract, original title, name of substance word, subject heading word, floating sub-heading word, keyword heading word, organism supplementary concept word, protocol supplementary concept word, rare disease supplementary concept word, unique identifier, synonyms]

#3 #1 or #2

#4 exp Heart Arrest/

#5 exp Ventricular Fibrillation/

#6 exp Cardiopulmonary Resuscitation/

#7 exp Heart Massage/

#8 (heart arrest or cardiac arrest or cardiopulmonary arrest or asystole or ventricular fibrillation or pulseless* or heart massage or cardiac massage or chest compression or cardiopulmonary resuscitation or CPR or advanced cardiac life support or ACLS).mp. [mp=title, abstract, original title, name of substance word, subject heading word, floating sub-heading word, keyword heading word, organism supplementary concept word, protocol supplementary concept word, rare disease supplementary concept word, unique identifier, synonyms]

#9 #4 or #5 or #6 or #7 or #8

#10 exp Systematic Review/

#11 exp Meta-Analysis/

#12 (review* or meta-analys* or overview*).mp. [mp=title, abstract, original title, name of substance word, subject heading word, floating sub-heading word, keyword heading word, organism supplementary concept word, protocol supplementary concept word, rare disease supplementary concept word, unique identifier, synonyms]

#13 #10 or #11 or #12

#14 #3 and #9

#15 #13 and #14

**Search strategy (ProQuest Health and Medical Collection)**

#1

(MESH.EXACT.EXPLODE("Vasopressins:D.27.505.696.560.249.pa2") OR MESH.EXACT.EXPLODE("Vasopressins:D.27.505.954.411.793.pa5") OR MESH.EXACT.EXPLODE("Vasopressins:D.12.644.548.691.692.781") OR MESH.EXACT.EXPLODE("Vasopressins:D.12.776.631.650.937") OR MESH.EXACT.EXPLODE("Vasopressins:D.12.644.400.900") OR MESH.EXACT.EXPLODE("Vasopressins:D.12.644.456.925") OR MESH.EXACT.EXPLODE("Vasopressins:D.27.505.954.502.270.463.pa6") OR MESH.EXACT.EXPLODE("Vasopressins:D.06.472.699.631.692.781")) OR noft(vasopressin* or vasopressor* or antidiuretic hormone* or argipressin or pitressin)

#2

MESH.EXACT.EXPLODE("Heart Arrest") OR (MESH.EXACT.EXPLODE("Ventricular Fibrillation:C.23.550.073.922") OR MESH.EXACT.EXPLODE("Ventricular Fibrillation:C.14.280.067.922")) OR MESH.EXACT.EXPLODE("Cardiopulmonary Resuscitation") OR (MESH.EXACT.EXPLODE("Heart Massage:E.02.365.647.375") OR MESH.EXACT.EXPLODE("Heart Massage:E.04.100.376.458") OR MESH.EXACT.EXPLODE("Heart Massage:E.04.928.220.380")) OR noft(heart arrest or cardiac arrest or cardiopulmonary arrest or asystole or ventricular fibrillation or pulseless* or heart massage or cardiac massage or chest compression or cardiopulmonary resuscitation or CPR or advanced cardiac life support or ACLS)

#3

MESH.EXACT.EXPLODE("Systematic Reviews as Topic") OR (MESH.EXACT.EXPLODE("Meta-Analysis as Topic:E.05.318.370.500") OR MESH.EXACT.EXPLODE("Meta-Analysis as Topic:N.06.850.520.445.500") OR MESH.EXACT.EXPLODE("Meta-Analysis as Topic:E.05.581.500.501") OR MESH.EXACT.EXPLODE("Meta-Analysis as Topic:N.05.715.360.325.515")) OR noft(review* or meta-analys* or overview*)

#4 (#1 AND #2)

((MESH.EXACT.EXPLODE("Vasopressins:D.27.505.696.560.249.pa2") OR MESH.EXACT.EXPLODE("Vasopressins:D.27.505.954.411.793.pa5") OR MESH.EXACT.EXPLODE("Vasopressins:D.12.644.548.691.692.781") OR MESH.EXACT.EXPLODE("Vasopressins:D.12.776.631.650.937") OR MESH.EXACT.EXPLODE("Vasopressins:D.12.644.400.900") OR MESH.EXACT.EXPLODE("Vasopressins:D.12.644.456.925") OR MESH.EXACT.EXPLODE("Vasopressins:D.27.505.954.502.270.463.pa6") OR MESH.EXACT.EXPLODE("Vasopressins:D.06.472.699.631.692.781")) OR noft(vasopressin* OR vasopressor* OR antidiuretic hormone* OR argipressin OR pitressin)) AND (MESH.EXACT.EXPLODE("Heart Arrest") OR (MESH.EXACT.EXPLODE("Ventricular Fibrillation:C.23.550.073.922") OR MESH.EXACT.EXPLODE("Ventricular Fibrillation:C.14.280.067.922")) OR MESH.EXACT.EXPLODE("Cardiopulmonary Resuscitation") OR (MESH.EXACT.EXPLODE("Heart Massage:E.02.365.647.375") OR MESH.EXACT.EXPLODE("Heart Massage:E.04.100.376.458") OR MESH.EXACT.EXPLODE("Heart Massage:E.04.928.220.380")) OR noft(heart arrest OR cardiac arrest OR cardiopulmonary arrest OR asystole OR ventricular fibrillation OR pulseless* OR heart massage OR cardiac massage OR chest compression OR cardiopulmonary resuscitation OR CPR OR advanced cardiac life support OR ACLS))

#5 (#3 AND #4)

((MESH.EXACT.EXPLODE("Vasopressins:D.27.505.696.560.249.pa2") OR MESH.EXACT.EXPLODE("Vasopressins:D.27.505.954.411.793.pa5") OR MESH.EXACT.EXPLODE("Vasopressins:D.12.644.548.691.692.781") OR MESH.EXACT.EXPLODE("Vasopressins:D.12.776.631.650.937") OR MESH.EXACT.EXPLODE("Vasopressins:D.12.644.400.900") OR MESH.EXACT.EXPLODE("Vasopressins:D.12.644.456.925") OR MESH.EXACT.EXPLODE("Vasopressins:D.27.505.954.502.270.463.pa6") OR MESH.EXACT.EXPLODE("Vasopressins:D.06.472.699.631.692.781")) OR noft(vasopressin* OR vasopressor* OR antidiuretic hormone* OR argipressin OR pitressin)) AND (MESH.EXACT.EXPLODE("Heart Arrest") OR (MESH.EXACT.EXPLODE("Ventricular Fibrillation:C.23.550.073.922") OR MESH.EXACT.EXPLODE("Ventricular Fibrillation:C.14.280.067.922")) OR MESH.EXACT.EXPLODE("Cardiopulmonary Resuscitation") OR (MESH.EXACT.EXPLODE("Heart Massage:E.02.365.647.375") OR MESH.EXACT.EXPLODE("Heart Massage:E.04.100.376.458") OR MESH.EXACT.EXPLODE("Heart Massage:E.04.928.220.380")) OR noft(heart arrest OR cardiac arrest OR cardiopulmonary arrest OR asystole OR ventricular fibrillation OR pulseless* OR heart massage OR cardiac massage OR chest compression OR cardiopulmonary resuscitation OR CPR OR advanced cardiac life support OR ACLS)) AND (MESH.EXACT.EXPLODE("Systematic Reviews as Topic") OR (MESH.EXACT.EXPLODE("Meta-Analysis as Topic:E.05.318.370.500") OR MESH.EXACT.EXPLODE("Meta-Analysis as Topic:N.06.850.520.445.500") OR MESH.EXACT.EXPLODE("Meta-Analysis as Topic:E.05.581.500.501") OR MESH.EXACT.EXPLODE("Meta-Analysis as Topic:N.05.715.360.325.515")) OR noft(review* OR meta-analys* OR overview*))

**Search strategy (Scopus)**

#1

TITLE-ABS-KEY (vasopressin* OR vasopressor* OR "antidiuretic hormone*" OR argipressin OR pitressin)

#2

TITLE-ABS-KEY ("heart arrest" OR "cardiac arrest" OR "cardiopulmonary arrest" OR asystole OR "ventricular fibrillation" OR pulseless* OR "heart massage" OR "cardiac massage" OR "chest compression" OR "cardiopulmonary resuscitation" OR cpr OR "advanced cardiac life support" OR acls)

#3

TITLE-ABS-KEY (review* OR meta-analys* OR overview*)

#4 (#1 AND #2)

(TITLE-ABS-KEY (vasopressin* OR vasopressor* OR "antidiuretic hormone*" OR argipressin OR pitressin) AND TITLE-ABS-KEY ("heart arrest" OR "cardiac arrest" OR "cardiopulmonary arrest" OR asystole OR "ventricular fibrillation" OR pulseless* OR "heart massage" OR "cardiac massage" OR "chest compression" OR "cardiopulmonary resuscitation" OR cpr OR "advanced cardiac life support" OR acls))

#5 (#3 AND #4)

(TITLE-ABS-KEY (vasopressin* OR vasopressor* OR "antidiuretic hormone*" OR argipressin OR pitressin) AND TITLE-ABS-KEY ("heart arrest" OR "cardiac arrest" OR "cardiopulmonary arrest" OR asystole OR "ventricular fibrillation" OR pulseless* OR "heart massage" OR "cardiac massage" OR "chest compression" OR "cardiopulmonary resuscitation" OR cpr OR "advanced cardiac life support" OR acls ) AND TITLE-ABS-KEY (review* OR meta-analys* OR overview*))

**Search strategy (Web of Science)**

#1

vasopressin* OR vasopressor* OR antidiuretic hormone* OR argipressin OR pitressin (All Fields)

#2

heart arrest OR cardiac arrest OR cardiopulmonary arrest OR asystole OR ventricular fibrillation OR pulseless* OR heart massage OR cardiac massage OR chest compression OR cardiopulmonary resuscitation OR CPR OR advanced cardiac life support OR ACLS (All Fields)

#3

review* OR meta-analys* OR overview* (All Fields)

#4 (#1 AND #2)

vasopressin* OR vasopressor* OR antidiuretic hormone* OR argipressin OR pitressin (All Fields) and heart arrest OR cardiac arrest OR cardiopulmonary arrest OR asystole OR ventricular fibrillation OR pulseless* OR heart massage OR cardiac massage OR chest compression OR cardiopulmonary resuscitation OR CPR OR advanced cardiac life support OR ACLS (All Fields)

#5 (#3 AND #4)

vasopressin* OR vasopressor* OR antidiuretic hormone* OR argipressin OR pitressin (All Fields) AND heart arrest OR cardiac arrest OR cardiopulmonary arrest OR asystole OR ventricular fibrillation OR pulseless* OR heart massage OR cardiac massage OR chest compression OR cardiopulmonary resuscitation OR CPR OR advanced cardiac life support OR ACLS (All Fields) AND review* OR meta-analys* OR overview* (All Fields)

**Search strategy (Cochrane Library)**

#1 MeSH descriptor: [Vasopressins] explode all trees

#2 vasopressin* or vasopressor* or antidiuretic hormone* or argipressin or pitressin

#3 #1 or #2

#4 MeSH descriptor: [Heart Arrest] explode all trees

#5 MeSH descriptor: [Ventricular Fibrillation] explode all trees

#6 MeSH descriptor: [Cardiopulmonary Resuscitation] explode all trees

#7 MeSH descriptor: [Heart Massage] explode all trees

#8 heart arrest or cardiac arrest or cardiopulmonary arrest or asystole or ventricular fibrillation or pulseless* or heart massage or cardiac massage or chest compression or cardiopulmonary resuscitation or CPR or advanced cardiac life support or ACLS

#9 #4 or #5 or #6 or #7 or #8

#10 MeSH descriptor: [Systematic Review] explode all trees

#11 MeSH descriptor: [Meta-Analysis] explode all trees

#12 review* or meta-analys* or overview*

#13 #10 or #11 or #12

#14 #3 and #9

#15 #13 and #14

**Supplementary Information 3**

**AMSTAR 2: Appraisal tool**

| Item | Content | Option |
| --- | --- | --- |
| 1 | Did the research questions and inclusion criteria for the review include the components of PICO (population, intervention, comparator group, outcome)? | Yes, No |
| 2* | Did the report of the review contain and explicit statement that the review methods were established prior to conduct of the review and did the report justify any significant deviations from the protocol? | Yes, Partial yes, No |
| 3 | Did the review authors explain their selection of the study for inclusion in the review? | Yes, No |
| 4* | Did the review authors use a comprehensive literature search strategy? | Yes, Partial yes, No |
| 5 | Did the review authors perform study selection in duplicate? | Yes, No |
| 6 | Did the review authors perform data extraction in duplicate? | Yes, No |
| 7* | Did the review authors provide a list of excluded studies and justify the exclusions? | Yes, Partial yes, No |
| 8 | Did the review authors describe the included studies in adequate detail? | Yes, Partial yes, No |
| 9* | Did the review authors use a satisfactory technique for assessing risk of bias (RoB) in individual studies that were included in the review? | Yes, Partial yes, No |
| 10 | Did the review authors report on the sources of funding for the studies included in the review? | Yes, No |
| 11* | If meta-analysis was performed, did the review authors use appropriate methods for statistical combination of results? | Yes, No, No meta-analysis conducted |
| 12 | If meta-analysis was performed, did the review authors assess the potential impact of RoB in individual studies on the results of the meta-analysis or other evidence synthesis? | Yes, No, No meta-analysis conducted |
| 13* | Did the review authors account for RoB in individual studies when interpreting/discussing the results of the review? | Yes, No |
| 14 | Did the review authors provide a satisfactory explanation for, and discussion of, any heterogeneity observed in the results of the review? | Yes, No |
| 15* | If they performed quantitative synthesis, did the review authors carry out an adequate investigation of publication bias (small study bias) and discuss its likely impact on the results of the review? | Yes, No, No meta-analysis conducted |
| 16 | Did the review authors report any potential sources of conflict of interest, including any funding they received for conducting the review? | Yes, No |

* Critical domains

**AMSTAR 2: Critical domains**

| Item | Explanation |
| --- | --- |
| 2 | Protocol registered before commencement of the review |
| 4 | Adequacy of the literature search |
| 7 | Justification for excluding individual studies |
| 9 | Risk of bias (RoB) from individual studies being included in the review |
| 11 | Appropriateness of meta-analytical methods |
| 13 | Consideration of RoB when interpreting the results of the review |
| 15 | Assessment of presence and likely impact of publication bias |

**AMSTAR 2: Rating overall confidence in the results of the review**

| Rating | Explanation |
| --- | --- |
| High | No or one non-critical weakness:  The systematic review provides an accurate and comprehensive summary of the results of the available studies that address the question of interest. |
| Moderate | More than one non-critical weakness*:  The systematic review has more than one weakness but no critical flaws. It may provide an accurate summary of the results of the available studies that were included in the review. |
| Low | One critical flaw with or without non-critical weaknesses:  The review has a critical flaw and may not provide an accurate and comprehensive summary of the available studies that address the question of interest. |
| Critically low | More than one critical flaw with or without non-critical weaknesses:  The review has more than one critical flaw and should not be relied on to provide an accurate and comprehensive summary of the available studies. |

* Multiple non-critical weaknesses may diminish confidence in the review and it may be appropriate to move the overall appraisal down from moderate to low confidence.

**Supplementary Information 4**

**List of studies excluded after a full-text review**

| **Author** | **Year** | **Title** | **Reason for exclusion** |
| --- | --- | --- | --- |
| Botnaru, T., Altherwi, T., & Dankoff, J. | 2015 | Improved neurologic outcomes after cardiac arrest with combined administration of vasopressin, steroids, and epinephrine compared to epinephrine alone | Not the desired study design |
| Desclefs, J. P. | 2018 | Vasopressors during the cardiopulmonary resuscitation. A meta-analysis of randomized trials | Not written in English |
| Jochberger, S., Wenzel, V., & Dünser, M. W. | 2005 | Arginine vasopressin as a rescue vasopressor agent in the operating room | Not the desired study design |
| Koshman, S. L., Zed, P. J., & Abu-Laban, R. B. | 2005 | Vasopressin in cardiac arrest | Not the desired study design |
| Lundin, A., Djärv, T., Engdahl, J., Hollenberg, J., Nordberg, P., Ravn-Fischer, A., Ringh, M., Rysz, S., Svensson, L., Herlitz, J., & Lundgren, P. | 2016 | Drug therapy in cardiac arrest: A review of the literature | Not the desired study design |
| Ong, M. E. H., Lim, S. H., & Anantharaman, V. | 2002 | Intravenous adrenaline or vasopressin in sudden cardiac arrest: A literature review | Not the desired study design |
| Srisurapanont, K., Thepchinda, T., Kwangsukstith, S., Saetiao, S., Kasirawat, C., Janmayka, W., & Wongtanasarasin, W. | 2021 | Comparing drugs for out-of-hospital, shock-refractory cardiac arrest: Systematic review and network meta-analysis of randomized controlled trials | Not the desired population |
| Vandersmissen, H., Gworek, H., Dewolf, P., & Sabbe, M. | 2021 | Drug use during adult advanced cardiac life support: An overview of reviews | Not the desired study design |
| Wyer, P. C., Perera, P., Jin, Z., Zhou, Q., Cook, D. J., Walter, S. D., & Guyatt, G. H. | 2006 | Vasopressin or epinephrine for out-of-hospital cardiac arrest | Not the desired study design |
| Xanthos, T., & Papadimitriou, D. | 2008 | Vasopressors in cardiopulmonary resuscitation | Not written in English |
